# Determinants of user acceptance of a specific social platform for older adults: An empirical examination of user interface characteristics and behavioral intention

**DOI:** 10.1371/journal.pone.0180102

**Published:** 2017-08-24

**Authors:** Tsai-Hsuan Tsai, Hsien-Tsung Chang, Yan-Jiun Chen, Yung-Sheng Chang

**Affiliations:** 1 Department of Industrial Design, College of Management, Chang Gung University, Taoyuan, Taiwan; 2 Division of Cardiology, Department of Internal Medicine, Chang Gung Memorial Hospital, Linkou, Taoyuan, Taiwan; 3 Department of Visual Communication Design, Ming Chi University, New Taipei City, Taiwan; 4 Department of Computer Science and Information Engineering, College of Engineering, Chang Gung University, Taoyuan, Taiwan; 5 Department of Physical Medicine and Rehabilitation, Chang Gung Memorial Hospital, Linkou, Taoyuan, Taiwan; Dalian University of Technology, CHINA

## Abstract

The use of the Internet and social applications has many benefits for the elderly, but numerous investigations have shown that the elderly do not perceive online social networks as a friendly social environment. Therefore, TreeIt, a social application specifically designed for the elderly, was developed for this study. In the TreeIt application, seven mechanisms promoting social interaction were designed to allow older adults to use social networking sites (SNSs) to increase social connection, maintain the intensity of social connections and strengthen social experience. This study’s main objective was to investigate how user interface design affects older people’s intention and attitude related to using SNSs. Fourteen user interface evaluation heuristics proposed by Zhang et al. were adopted as the criteria to assess user interface usability and further grouped into three categories: system support, user interface design and navigation. The technology acceptance model was adopted to assess older people’s intention and attitude related to using SNSs. One hundred and one elderly persons were enrolled in this study as subjects, and the results showed that all of the hypotheses proposed in this study were valid: system support and perceived usefulness had a significant effect on behavioral intention; user interface design and perceived ease of use were positively correlated with perceived usefulness; and navigation exerted an influence on perceived ease of use. The results of this study are valuable for the future development of social applications for the elderly.

## Introduction

In the face of an aging population, many researchers have recently begun to pay attention to the psychological well being and social needs of older people. A social network refers to the network-type structure formed among people or between people and organizations through connections such as interpersonal relations, social relations, etc., which can range from close to distant [1]. Given a certain stage of life and living environment, individuals, in particular older people after retirement, respond rather differently to social networks. Because of environmental restrictions, seniors who once spent most of their time at work and established social interactions in their workplaces often find it difficult, once retired, to expand the scope of their social lives and maintain their earlier social relations. Furthermore, age-related deterioration of physiological functions and senses limit opportunities for social contact. In addition to aging and retirement, the elderly must cope with grief resulting from widowhood and the loss of loved ones and friends, leading to loneliness and a lack of social contact and interaction [2,3]. Many studies have suggested that social relations and social interactions are closely associated with maintaining physiological function and mental health in the elderly. For instance, poor social relationships increase the risk of coronary heart disease in the elderly [4]. Social interactions and social activities can remedy the social isolation, loneliness, depression and cognitive impairment of older people [5,6]. Social activities can maintain the physiological function of elderly people and help reduce the risk of Alzheimer's disease [7]. Social interaction has the same effect as staying fit on reducing the risk of death [8]. As they age, older people are more willing to spend time maintaining connections with family members and close friends [9]. Emotional connection and closeness with family members and loved ones make older people feel happy [10], and social interaction with friends helps older people achieve a sense of happiness and intimacy, moderate their self-disclosure and emotional support, and maintain their self-worth [11]. Therefore, social relations and social conditions may affect the psychological and physical health of the elderly. Thus, to promote the health of the elderly, their social life must also be promoted.

Generally, the elderly are assumed not to be adept at digital technology, but this is not actually the case. As younger generations face changes in life stage and social scope, for instance during the transition from high school to college, they use social networking sites (SNSs) to connect with past and present social relationships. The elderly behave the same way [12]. According to a 2014 Pew Research Center survey, in recent years approximately 56% of the population over the age of 65 has used Facebook [13]. In the United States, only 13% of elderly Internet users had used SNSs in 2009, but the proportion had increased to 43% by 2013 [14,15]. In addition, the survey noted that 1 in 3 (34%) SNS users over 65 used Facebook and that 18% of elderly social network users used various SNSs on a daily basis [15]. Many studies have shown that whether older adults used SNSs, Skype or blogs, networking skills enabled them to strengthen social ties. Furthermore, the motivation that prompted the elderly to want to learn communication technology was often the desire either to stay in touch with family members and friends in distant locations or to have more contact with younger generations within the family. Therefore, SNSs have become a viable tool for connecting with family and friends and expanding social reach.

A variety of social interaction applications have been designed specifically for the elderly. For example, PawPawMail is an iPad application first developed by QPQ Analytics in 2011 [16]. It is an email system specifically designed for the elderly, in which users do not have to log in to an email account and the screen only displays basic information, buttons and images. In addition, it emphasizes use by both the elderly person and the caregiver via an interoperable email account, so the caregiver can assist in setting up a contact book, deleting junk emails and sending pictures to the elderly person’s children. Similarly, Keep-in-Touch is a communication application for the elderly first launched on July 9, 2012 [17]. It only contains functions suitable for the elderly to receive and send voice messages and to view and send pictures. Although PawPawMail and Keep-in-Touch provide clear and simple functionality, user interface and communication functions, they have not been validated in terms of acceptance and usability by the elderly. Furthermore, the design of social networking sites currently does not target the elderly nor provide a user interface that can perform a social function meeting the habits and needs of the elderly. To create a different social network experience, maintain familiar social patterns and enhance the strength of social connections for older adults, we designed a social application called TreeIt. TreeIt applies Granovetter’s strength of social ties theory [18] and Gilbert’s formula calculating the strength of social connection through social media [19]. It also applies Dunbar’s number theory [20] to limit the social network size to 150 people to establish a circle of close friends and maintain effective interaction. Based on these design concepts and social theories, seven social networking factors as well as the equation for calculating strength of connection were applied. In addition to defining the connection value of each user in a social circle, the contacts with the highest frequency of interaction with the user were identified. The “create,” “maintain” and “enhance,” mechanisms to promote social interaction and strengthen social networking connections were also proposed. In addition, mobile devices and applications were employed to provide older adults with convenient and real-time social interaction services. With respect to interface design, graphical user interface design and visualization of the user's social situation enhanced the online social experience and the willingness of older people to use TreeIt. Overall, in this study, we aimed to verify whether older adults were able to accept and use the TreeIt system. We also investigate the requirements and intention of older adults with regard to using these social network services.

### Heuristic evaluation and users’ technology acceptance of social technology

Users interact with a system through a user interface. To users, the user interface constitutes the totality of the system [21]. Evaluating the usability of a user interface is the key in system design and development because in so doing, it is possible to discover system errors and poor user interface design, enabling necessary improvements. Among many evaluation methods, the heuristic evaluation method has been the most commonly used [22,23]. It was put forward by Nielsen, and after a period of development, ten usability heuristics were formally proposed in 1995 [24]. Currently, heuristic evaluation checklists developed in studies on system usability evaluation are based on the ten heuristics proposed by Nielsen as evaluation items [25–27]. Based on Nielsen’s ten usability heuristics and Shneiderman and Plaisant’s eight Golden Rules of Interface Design [28], Zhang et al. [29] proposed 14 heuristics, adding items such as “informative feedback,” “clear closure,” “reversible actions” and “use user's language.” The four additional items were originally applied to determine the safety and usability of the 1-channel volumetric infusion pump used by patients. It is worth mentioning that many studies have adopted the 14 heuristics proposed by Zhang et al. to assess the usability of a system user interface [30–32].

Many researchers use heuristic evaluation (HE) to conduct usability assessments of social applications. Al-Badi et al. [33] conducted expert evaluation and user testing using HE to assess the usability of LinkedIn and detect its usability problems. In user testing, subjects were tested using the think-aloud protocol and observation to understand user behaviors. In the expert evaluation, Nielsen's ten usability heuristics were subdivided into a checklist to perform the system usability evaluation. Ashraf and Raza [34] investigated the usability of social applications for blind people. Because blind people encounter various challenges navigating web pages, the usability of social media such as YouTube and Facebook was investigated from their perspective using Nielsen's ten usability heuristics. Based on online and virtual reality technologies, Castilla, et al. [35] developed the Butler multiple software system, containing email, video, blog, browsing, image and sound galleries, a virtual reality environment, etc., to assist the elderly in using technology products via new forms of communication. The study included four stages of systematic assessment. In the last stage, experts were invited to perform a heuristic evaluation in which the ten usability heuristics were sub-divided into 69 sub-heuristics. The results of the expert evaluation showed that the system failed to meet five heuristics, of which four were important. Overall, the heuristic evaluation method has been extensively used in usability evaluations of social software and social technology. Based on the goal of this study and the characteristics of the TreeIt system, we adopted the 14 heuristics proposed by Zhang, et al. [29] to perform a usability evaluation of the use of the TreeIt system by older adults.

In the field of information technology, user intention and attitude are crucial issues. The technology acceptance model (TAM) [36] has been widely used to develop application tools that can evaluate and predict whether an information system or information technology will be accepted by users. The TAM has been used in previous studies as a common analytical framework to investigate users’ behavior with regard to using new technologies. The key advantage of the TAM is that it has a systematic quantitative analysis model and rich experience accumulated from being used to evaluate the acceptance of social network sites. For example, Yang and Lin [37] used the TAM2 to investigate the willingness of employees in a Taiwanese manufacturing company to use SNSs as a supplementary learning tool. The results showed that in addition to validating the technology acceptance model, the TAM2 confirmed not only that social influence could predict perceived usefulness but also that perceived ease of use was affected by computer self-efficacy and perceived enjoyment and concentration. Hu et al. [38] used the TAM2 to investigate SNS acceptance of non-social network users, and the results confirmed that perceived enjoyment and perceived social norms exerted a significant influence on behavioral intentions. Choi and Chung [39] studied the impact of social norms and perceived social capital on users’ acceptance and use of SNSs. The results demonstrated that subjective norms and perceived social capital could significantly predict perceived usefulness and perceived ease of use of SNSs by users. Rauniar et al. [40] proposed the “Revised Social Media TAM” to investigate users’ attitude and behavior with regard to using SNSs. Their model supplemented the TAM previously proposed by Davis et al. with “critical mass,” “capability,” “perceived playfulness” and “trustworthiness.” The results showed that all hypotheses had a significant impact on the use of SNSs. Moreover, a number of studies specifically used the TAM to investigate older adults’ behavior and attitude with regard to using SNSs. For example, Braun [41] examined which factors fostered or hampered older adults’ intention and motivation to use SNSs and found that frequency of Internet use, perceived usefulness and trust in SNSs could significantly predict intention to use SNSs. Pan and Jordan-Marsh [42] conducted a similar study and found that perceived usefulness, perceived ease of use, subjective norms and facilitating conditions could predict older Chinese adults’ intention to use SNSs. Tsai et al. [43] developed the Memotree system to facilitate social interactions among family members by prompting intergenerational communication. To verify the usability of the Memotree system, the TAM was adopted to promote user acceptance of the proposed Family Communication Application. The results indicated that technology affordances and perceived ease of use have a positive impact on perceived usefulness, whereas perceived ease of use is affected by technology affordances. Internet self-efficacy and perceived usefulness have a positive impact on the user’s behavioral intention toward the system. Given that the TAM has previously been used in the investigation and analysis of SNS technology use behavior and has shown strong explanatory power, it was adopted in this study to investigate older adults’ intention and behavior related to using the TreeIt system, a mobile social networking application.

### Statistical studies on the relationship between user interface usability and the TAM

In the field of usability research, a number of scholars have used statistical models to investigate the relationships among user interface (UI), usability and the TAM. Nikov et al. [44] proposed structural equation modeling (SEM) for web service usability, aiming both to develop checklists suitable for assessing the usability and quality of web services and to determine the effect of objective measurements of web service usability on subjective measurements, including “SERVQUAL,” “usability heuristic” and “ISO 9241–10.” The results showed that for web services, quality was very similar to usability and the objective measurements of web service usability strongly and significantly influenced the subjective measurements of usability. Oztekin, et al. [45] proposed the UWIS usability evaluation method by combining the quality and usability aspects of a web-based information service and found that the quality and usability of the web-based information system were significantly correlated. Oztekin et al. [46] then developed UseLearn, a usability assessment checklist for an eLearning system, which contained quality and usability evaluation items and used the structural equation modeling method to verify the checklist. The results showed that the quality and usability of the eLearning system were closely correlated. In addition to the relationship between system quality and usability, a number of researchers conducted investigations on the relationship between system user interface usability and TAM factors. Calisir and Calisir [47] investigated the relationship between user interface and perceived usefulness or perceived ease of use of an enterprise resource planning (ERP) system. They found that perceived ease of use and system capability exerted a direct influence on perceived usefulness and an indirect influence on end-user satisfaction through perceived usefulness. Moreover, user guidance had a direct impact on perceived usefulness and learnability had an indirect impact on end-user satisfaction. Cho et al. [48] assessed the effect of perceived user interface design (PUID) on user intention to continue using a self-paced eLearning tool and found that PUID had a direct impact on perceived usefulness and an indirect impact on perceived usefulness through perceived ease of use. In terms of continued usage intention, perceived usefulness and user satisfaction were important factors. However, in these studies, either the definitions of usability and user interface were too loose or only certain elements of Nielsen’s usability heuristics were adopted. The influence of user interface design on users’ attitudes or intention with regard to using a system was not fully investigated. In this study, in addition to adopting the 14 usability heuristics proposed by Zhang et al. [29] as evaluation criteria, the TAM proposed by Davis et al. [36] was used to evaluate the intention to use SNSs among older adults.

## Hypothesis development

**Fig 1:**
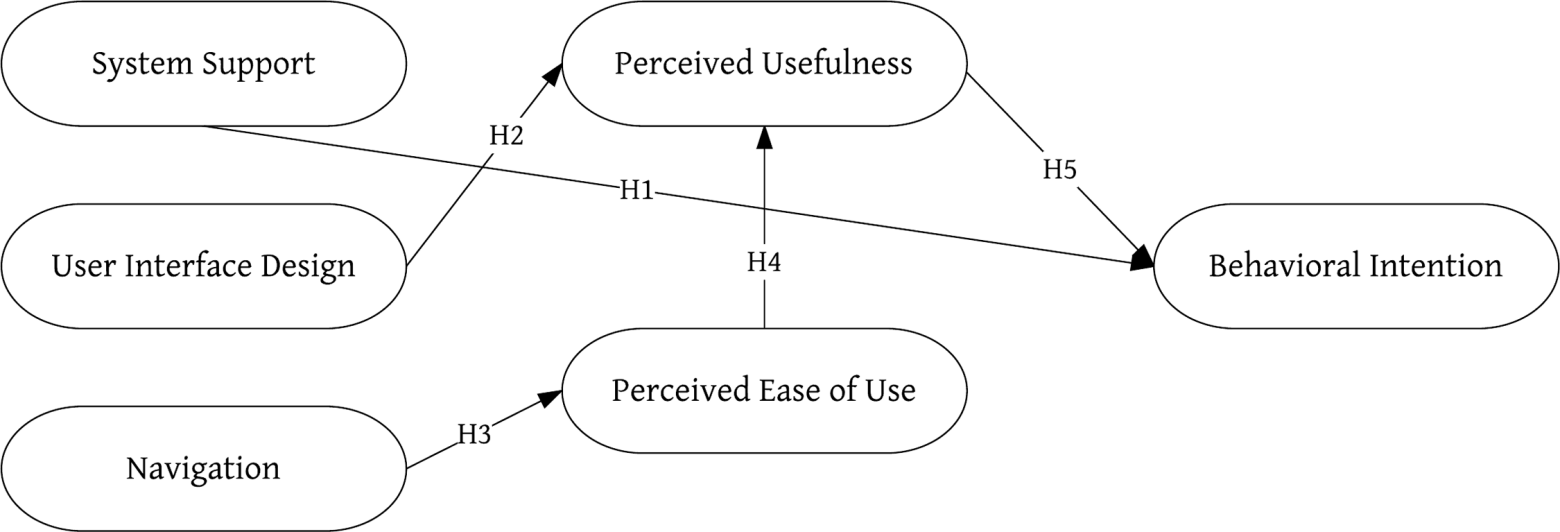
TreeIt research structure.

To understand how user interface affects older adults’ intention and attitude with regard to using TreeIt, a social platform, the 14 usability heuristics proposed by Zhang et al. [29] were adopted, namely, “H1 Consistency”, “H2 Visibility”, “H3 Match”, “H4 Minimalist”, “H5 Memory”, “H6 Feedback”, “H7 Flexibility”, “H8 Message”, “H9 Error”, “H10 Closure”, “H11 Undo”, “H12 Language”, “H13 Control”, and “H14 Document”. However, because the 14 heuristics included several overlapping features, they were grouped into three factors (system support, user interface design and navigation) based on previous studies [46,48,49] (Table 1) and was assessed by five suitable experts. The other three factors (perceived ease of use, perceived usefulness and behavioral intention) were adopted from the TAM. The definition of each factor and hypotheses regarding the relationship between the factors are described below, while the study’s research framework is shown in Fig 1.

**Table 1:** Categorization of Zhang et al.'s usability heuristics into three groups.

Category	System support	User-interface design	Navigation
Zheng et al.’s Heuristic Evaluation	H7. Flexibility	H1. Consistency	H3. Match
	H8. Message	H2. Visibility	H4. Minimalist
	H9. Error	H6. Feedback	H5. Memory
	H11. Undo	H10. Closure	H14. Document
	H12. Language	H13. Control	

### (1) System support

When using technology products or computers, older adults are often anxious because of a lack of experience or fear of negative outcomes due to operational errors [50,51]. Previous studies have shown that when using the Internet and mobile phones, older adults often experience anxiety [52]. Igbaria and Iivari [53] found that when feeling anxious about computers, users were likely to be unwilling to use them. However, when a technology product possesses a good support system, it can help users avoid making errors when operating the product and thus enhance users’ intention to operate the product. Cho et al. [48] defined system support as “the perceived effectiveness of system support for a system.” Wilkinson et al. [54] also defined perceived system support as “the technical and customized support to access the needed information without problems.” Based on previous definitions, in this study system support was defined as “the perceived effectiveness of system support at preventing and recovering from errors”. It included five usability heuristics, namely, “H7. Flexibility,” “H8. Message,” “H9. Errors,” “H11. Undo” and “H12. Language. The following hypothesis was proposed:

**H1.** System support is positively associated with behavioral intention to use the TreeIt system among older adults.

### (2) User interface design

A good user interface design allows user to operate specific functions on a system in different ways while being able to enhance the usefulness of the system [55]. User interface design, which can also be called screen design [56], is the visual appearance of a system, such as the arrangement of content in terms of layout, color schemes, icons, buttons and font sizes [57]. Based on this, five usability heuristics, i.e., “H1. Consistency,” “H2. Visibility,” “H6. Feedback,” “H10. Closure” and “H13. Control” were grouped under user interface design in this study. In addition, Cho et al. [48] demonstrated that when users operated the e-learning tool in the study, perceptive user interface design exerted a significant effect on perceived usefulness. Similarly, Hong et al. [58] indicated that in using the e-Library system under consideration, perceived usefulness could be significantly predicted based on screen design. Therefore, the following hypothesis was proposed:

**H2.** User interface design is positively associated with perceived usefulness of using the TreeIt system among older adults.

### (3) Navigation

If a system provides the appropriate navigation features, it should allow users to easily access the information they need from the system’s user interface. Dillon [59] found that inappropriate navigation features tended to make users feel disoriented when looking for information in the system, which was also confirmed by Marchionini et al. [60], who showed that the main cause of disorientation in users was that navigation within complex system frameworks increased the user’s cognitive load. Using the navigation of online libraries as an example, they demonstrated that by providing navigation aids or adding unique landmarks, the system could allow users to navigate more efficiently. In similar investigations, González et al. [49] defined “simple navigation” as “minimalist,” whereas Oztekin et al. [45,46] categorized “memory” as navigation and deemed “match” and “document” helpful to system navigation. Based on the aforementioned studies, four usability heuristics, i.e., “H3. Match”, “H4. Minimalist”, “H5. Memory”, and “H14. Document” were grouped under navigation in this study. Moreover, Ramayah [61] noted that system navigation could significantly affect perceived ease of use. Thus, the following hypothesis was proposed:

**H3.** Navigation is positively associated with perceived ease of use in using the TreeIt system among older adults.

### (4) Perceived ease of use, perceived usefulness, behavioral intention

Based on the definition of TAM by Venkatesh and Davis [62], Rauniar et al. [40] proposed applying the TAM to social technology and suggested that perceived ease of use should be defined as “the degree to which a person believes that using a particular system would be free of effort,” while perceived usefulness should be defined as “the extent to which the social media user believes that using a particular social media site help to meet the related goal-driven needs of the individual.” The relationships among perceived ease of use, perceived usefulness and behavioral intention have been verified by studies in many different areas. For instance, King and He [63] conducted a meta-analysis of 88 TAM-related studies and found that perceived usefulness and intention to use were significantly correlated. Many studies have shown that perceived ease of use has an impact on perceived usefulness. For example, Rauniar et al. [40] investigated which factors affected Facebook users’ attitude and acceptance of SNSs and found not only that when social networking sites were perceived as easy to use, users perceived those sites as useful but also that perceived usefulness and intention to use were significantly correlated. Therefore, we proposed the following hypotheses:

**H4.** Perceived ease of use is positively associated with perceived usefulness in using the TreeIt system among older adults.

**H5.** Perceived usefulness is positively associated with behavioral intention in using the TreeIt system among older adults.

## Methods

### Sampling and survey administration

The purpose of this study was to investigate whether user interface design affected older adults’ behavior with regard to social media use via the TreeIt system specifically developed for this study. A total of 101 older adults residing in New Taipei City, Taiwan, were recruited. Participant selection criteria were as follows: (a) over 50 years of age; (b) no structural heart disease; (c) capable of reading Mandarin Chinese; and (d) passed the Mini Mental State Examination (MMSE) with a score of over 24, indicating participants have strong mental abilities in areas such as memory, attention and language [64]. Ethical approval was obtained from the Institutional Review Board of Chang Gung Hospital, Taoyuan, Taiwan (104-5645C). All relevant ethical safeguards were met in relation to ethical consideration and subject protection.

The experiment was conducted at the New Taipei City Association for Senior Fellowship Center, Taiwan, where experimental facilities were set up in space provided by the association. The subjects were recruited by contacting the New Taipei City Association for Senior Fellowship Center, which was informed of the purpose, procedure and steps and subject qualifications of the study. After obtaining the consent and support of the Association, 101 subjects who met the requirements were recruited two weeks before the inception of the experiment. As shown in Table 2, 25.8% of the participants were male, whereas 74.2% were female. In terms of age, 6.9% were between 50 and 59; 64.4% were between 60 and 69; 24.8% were between 70 and 79; and 3.9% were between 80 and 89. As for education, 3% had graduated from a graduate school; 34.7% had graduated from a university; 41.6% had completed high school as their highest level of education; 6% had completed junior high school as their highest level of education; and 14.7% had completed elementary school as their highest level of education. Of the participants, 45.5% had previous experience using SNSs, whereas 54.5% of the participants had no SNS experience.

**Table 2:** Descriptive statistics of participants' characteristics.

Gender	N	%	Age	N	%	Education	N	%	SNS Experience	N	%
Female	75	74.2	50~59	7	6.9	Master	3	3.0	Yes	46	45.5
Male	26	25.8	60~69	65	64.4	University	35	34.7	No	55	54.5
Total	101	100.0	70~79	25	24.8	High school	42	41.6	Total	101	100
			80~89	4	3.9	Junior high school	6	6.0			
			Total	101	100.0	Elementary school	15	14.7			
						Total	101	100.0			

The experimental tools of this study were the TreeIt system, the subject consent form, the background information questionnaire, the TAM questionnaire, and the heuristic evaluation checklist. A 10.1-inch tablet computer was used to operate TreeIt. Other experimental recording tools such as laptop computers, digital cameras, video recorders, and audio recorders were also used. In addition, the TreeIt system used Facebook as the data source. Therefore, in terms of retrieving social interaction records data, we had to obtain the subjects’ basic information and activity records through the Facebook API, thus excluding the portion not deemed open access by Facebook and information the users had not agreed to disclose, e.g., friends lists, mobile phone numbers, addresses, etc. After the investigator explained the purpose of the experiment and the subject stated that he or she clearly understood the experiment, the subject was asked to sign the consent form. Because we were collecting participants’ information, the consent form strongly emphasized that no personal information would be disclosed. After the subject signed the consent form, the investigator asked the subject to log into his or her personal Facebook account via the computer so that the TreeIt system would collect any basic information and activity records the subject had disclosed on Facebook. The participant was asked to press the “Agree” button in the system, thereby granting permission for the Facebook API extraction. This process lasted approximately 5–10 min. After the TreeIt system synchronously updated the user's Facebook profile, the investigator introduced the system to the subject and asked the subject to operate it.

To enhance the reliability and validity of the experiment and achieve consistency in the experiment, standard operating procedure was carried out throughout the experiment. The procedure was to ask the participant to operate the following seven functions sequentially: (1) Social display function: this function graphically displayed the status of the user’s social interactions with his or her friends, i.e., it was a graphic presentation of one of the factors illustrating the strength of a relationship, namely, duration; (2) Chat function: this function provided suggested topics for the users to discuss with their friends. During this step, the subjects were asked to operate the chat function on the TreeIt system by selecting one online friend and opening the chat function interface; (3) Common album function: this function allowed users to quickly and accurately integrate photos with a common theme posted by their friends to an album. During this step, the subjects were asked to click and browse the common album; (4) Hot-topic function: in this step, the subjects were asked to click on the hot-topic function to browse the daily events most discussed by users and their friends; (5) Recent group function: this function showed the social status of the users and their friends. In this step, the subjects were asked to hold the “friends” leaf-shaped logo for a short length of time on the interface, which then displayed the list of friends with whom the user had most frequently communicated recently. This function provided a reference regarding the level of social interaction the user had had with them. Next, the user was asked to select one friend and press and hold that friend’s leaf to browse the recent group function; (6) Auto-suggest friends function: in this step, the investigator asked the user to add one friend based on the recommendation list made by the system; and (7) Mood display/emotional feedback function: in this step, the subjects were asked to select one friend according to his/her mood displayed by the system interface and to provide support and care to the friend through the function keys provided by the system interface. The purpose of the seven functions was to create, maintain, and enhance older adults’ social interactions with their friends using TreeIt. Auto-suggested friends and common album functions were categorized under the “create mechanism”. The social display and mood display/emotional feedback function were categorized under the “maintain” mechanism. Finally, the hot topic, recent group, and chat functions were categorized under the “enhance” mechanism. Each of the 101 elderly subjects completed the test accompanied by the investigator on a one-on-one basis. Then, the heuristic evaluation checklist and TAM questionnaire were filled out. The test for each subject lasted approximately 30 minutes.

### Material

The TreeIt system and its user interface used Granovetter's strength of social ties theory to determine the four factors of relationship strength: duration, predictive intensity, intimacy and reciprocal service [18]. Next it used Gilbert and Karahalios's measurable formula to predict the strength of connections among social nodes of SNSs. The accuracy of the formula in predicting the strength of the social network was increased by the factors of structural and social distance, which removed emotional support from predictive intensity and treated it as an independent factor so that users' invisible backgrounds could be taken into account [19,65]. Next, based on Dunbar's number theory, the number of a user's friends was limited to 150 to maintain effective interaction within the SNS [20]. Based on the proposed concept and applied theories, the contacts with the highest frequencies of interaction with the user were identified. In addition, the TreeIt application was designed to work on smart mobile devices, such as tablet PCs, thus providing flexibility and accessibility to elderly users who may not be familiar with computers and offering convenient and real-time social interaction services. The social status of the user is presented on the system interface as the growth status of a tree, in which each of the friends is portrayed as a leaf and interacts via the social functions provided by the system. By simulating the tree planting process, caring for each leaf is then akin to caring for each friend, while increasing the number of friends is akin to increasing the density of the leaves. In terms of functional design, TreeIt incorporated seven social promotion functions that allowed users to use SNSs to increase social connection, maintain the intensity of social connection and strengthen social experience. The descriptions of the seven functions under three mechanisms are as follows.

#### The “create” mechanism

**Fig 2:**
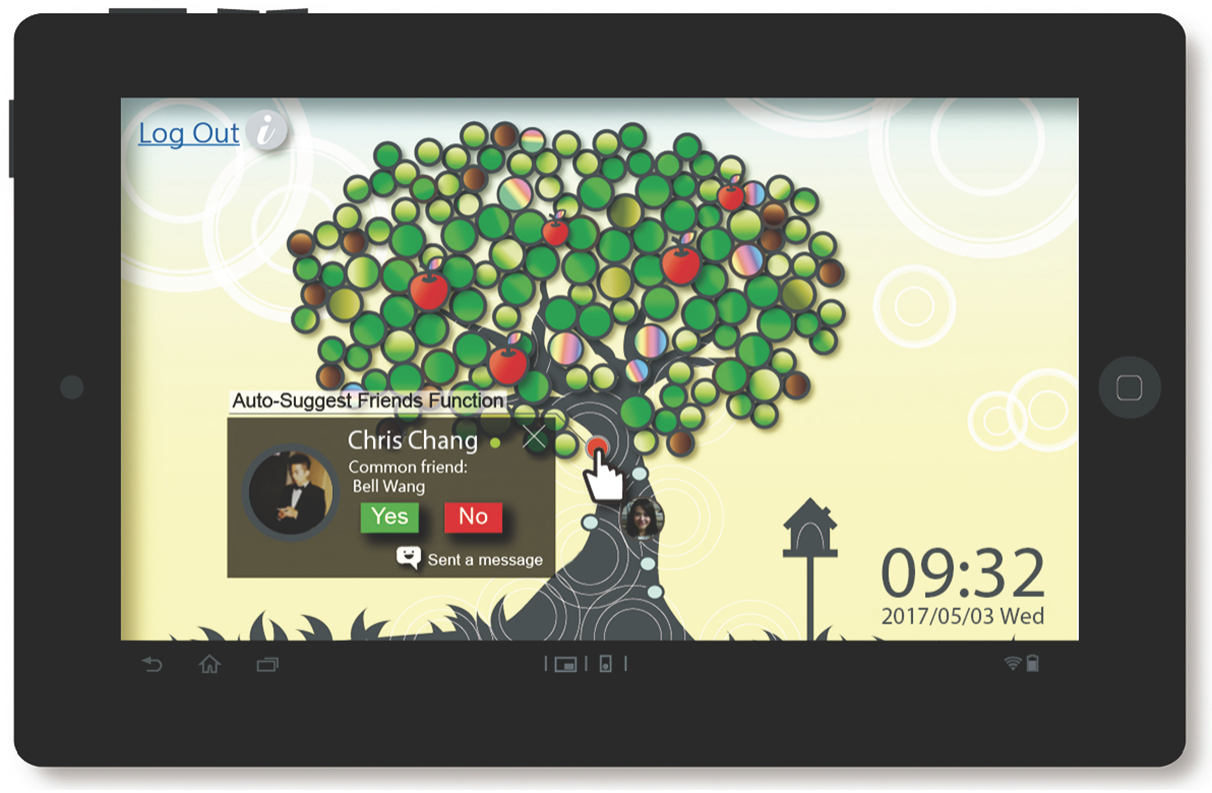
TreeIt system interface: Auto-suggest friends.

**Auto-suggest friends:** The main purpose of this function was to promote the generation of more connections with common links to help shorten social distance among friends. After the TreeIt system extracted the users’ social records and completed the analyses, it automatically generated a score based on a user’s tags, blogs, photos, number of check-ins and shared interests. If someone tagged was not a friend of the user (or vice versa), TreeIt would list this person as a potential friend. In the user interface design, auto-suggested friends were displayed as five buttons in the lower section of the system’s main screen, representing five potential friends recommended by the system. When the user clicked on a button, the system would provide the list of friends shared with the user as well as the tagging records of the auto-suggested friend. After the user agreed to add/refuse the auto-suggested friend, the system would recalculate and recommend another potential friend who shared common friends with the user (Fig 2).

**Fig 3:**
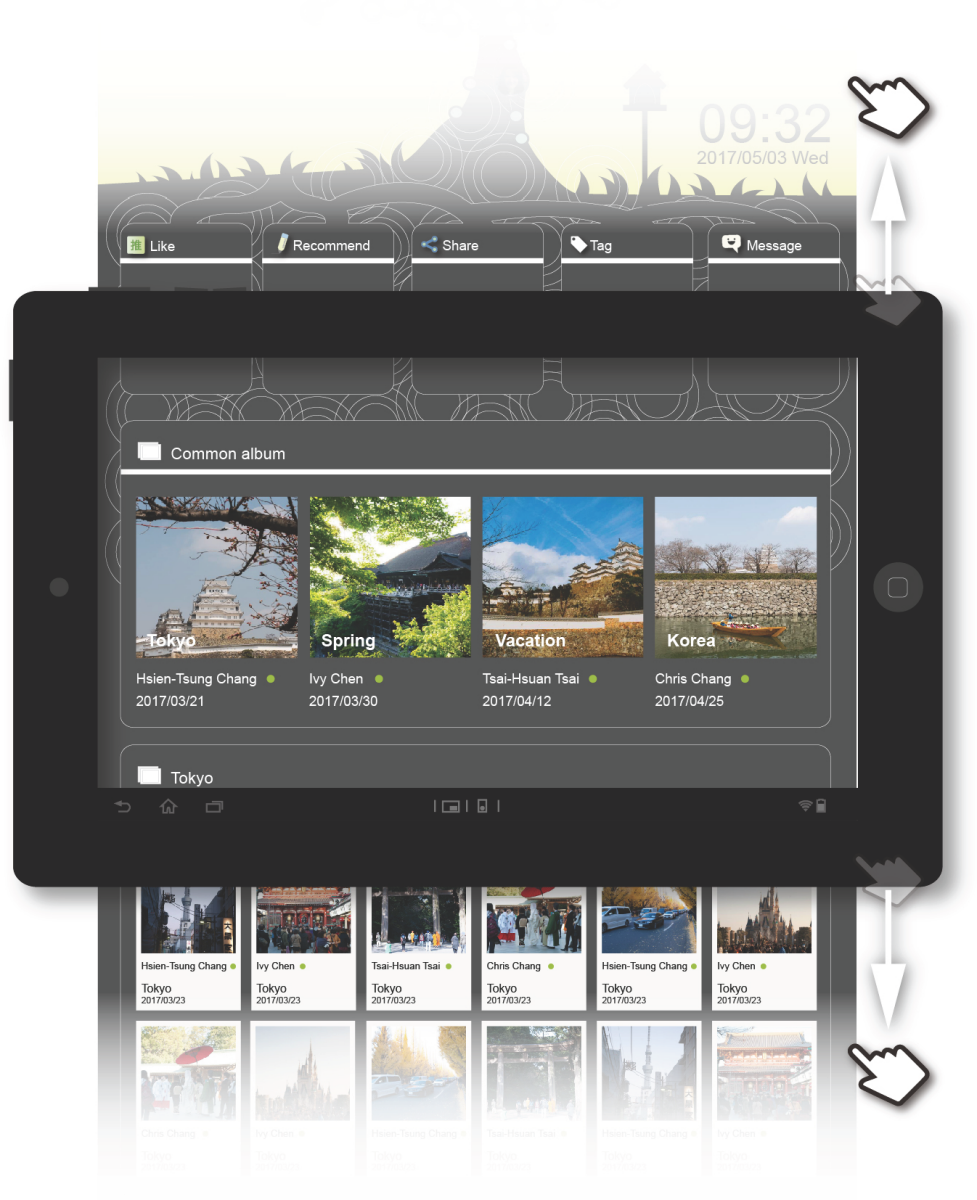
TreeIt system interface: Common album function.

**Common album function:** This feature helped users enhance intimacy with friends. When a user uploaded an album with the same theme as those uploaded by other friends, TreeIt displayed all of the friends' albums on the same page to make it easier for the user to view and collect them. In terms of user interface design, the common album function collected all the albums with the same theme into one collage. By clicking on an album with a specific theme, the user could simultaneously view the content of albums with a common theme by both user and friends. Under each photo, the source, date and time, description and location of the photo were presented (Fig 3).

#### The “maintain” mechanism

**Fig 4:**
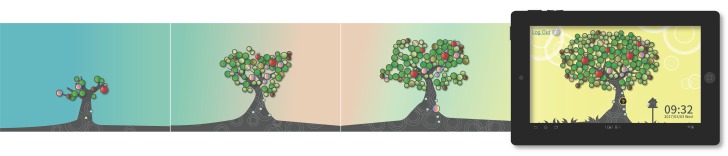
TreeIt system interface: Social display function.

**Social display function:** The main purpose of this function was to help users interact with friends. The TreeIt system retrieved information about total interaction time between the user and each friend from the first to the most recent interaction, including the number and content of the interactions. Based on the nature of the interactive functions the user used, the interactions were divided into two categories (unilateral use by the user and reciprocal use by the user and friends) and used to calculate the intensity of social connections. Based on this calculation, friends who had an above average intensity of social connection with the user were selected and displayed on the tree in their social status. When the intensity of social connection was higher than the average social index between the user and the user’s friends, it was considered a strong connection and represented by a large friend leaf, and vice versa. The main purpose of the social display function was to remind the user to engage in social interactions. Moreover, to maintain social intensity, the system automatically filtered out friends with whom the user had a weak connection, and only friends (up to 150) with an above-average social connection intensity were displayed on the interface (Fig 4).

**Fig 5:**
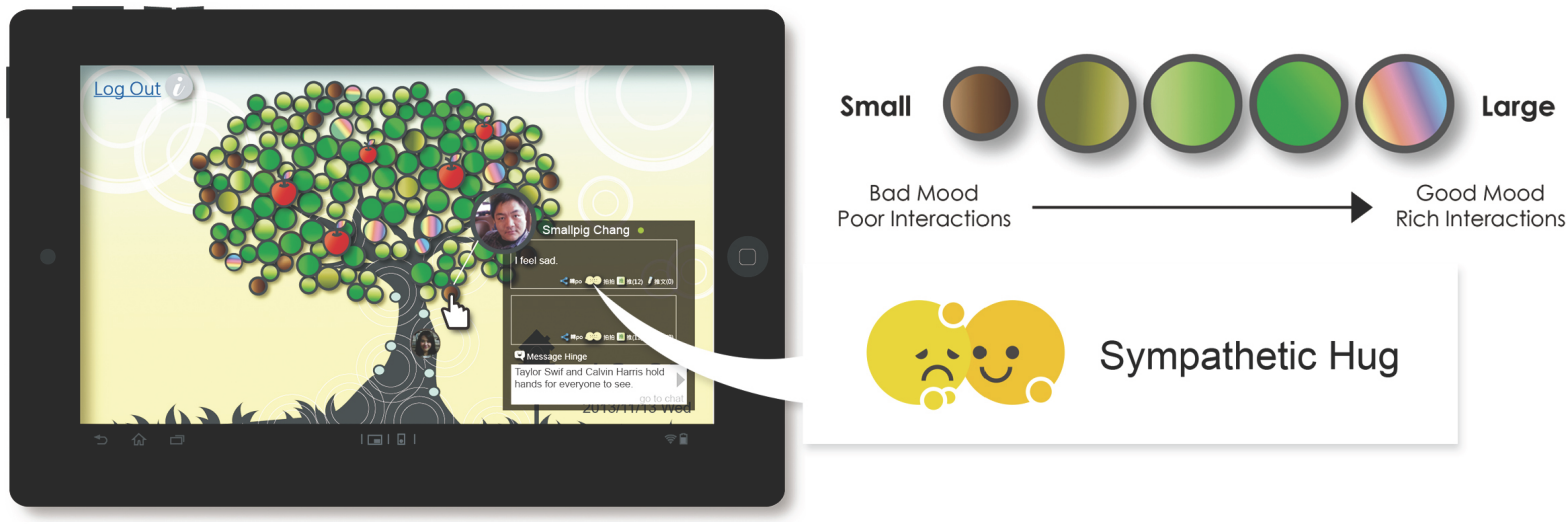
TreeIt system interface: Mood display/emotional feedback function.

**Mood display/emotional feedback function:** The main purpose of this function was to remind the user to provide appropriate care and emotional support for their friends. The TreeIt system can retrieve the dynamic status of the user’s friends and evaluate their most recent status. TreeIt can match the words in a friend’s status with the Chinese Word Sketch and evaluate positive or negative words appearing in the status. As a result, the system categorized the friends’ mood into positive or negative using five colors based on their status context. Then, based on the friend’s positive or negative mood, the user can provide emotional feedback by clicking on the relevant button to convey emotional support to the friend. When the friend accumulated or maintained a certain amount of emotional feedback, the color of the friend’s leaf either changed or remained the same (Fig 5).

#### The “enhance” mechanism

**Fig 6:**
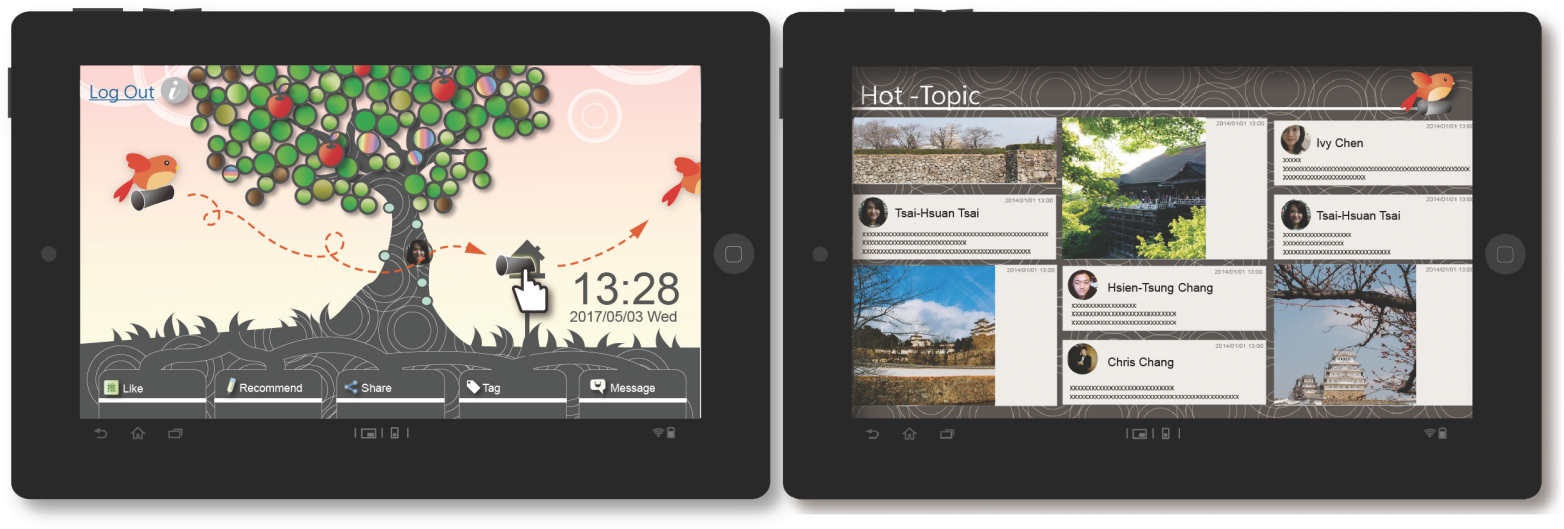
TreeIt system interface: Hot-topic function.

**Hot-topic function:** The main purpose of this function was to provide information regarding the most active discussions among the user and friends at a fixed time every day. An animated pigeon would remind the user to preferentially read the top trending news items among friends. Based on users and their friends’ most recent news posts and the number of likes and comments on the news items, the comments on the most-discussed messages (including all of the messages posted by the user and friends) were obtained and displayed on the hot-topic function interface. The hot-topic function regularly prompted and continuously updated the user interface. Every hour, on the left side of the system’s main screen, a flying pigeon appeared and sent a letter into the mailbox. The user was able to read the most popular news items among friends by clicking on the mailbox. In the one-hour period between system updates, the user could repeatedly click on the mailbox to read the most recent popular news items. Any new developments with respect to the most popular messages within the one-hour period would be displayed in real time until the next update, when the system would recalculate and arrange the previous hour’s hot topics and the user could obtain updated information by clicking on the mailbox (Fig 6).

**Fig 7:**
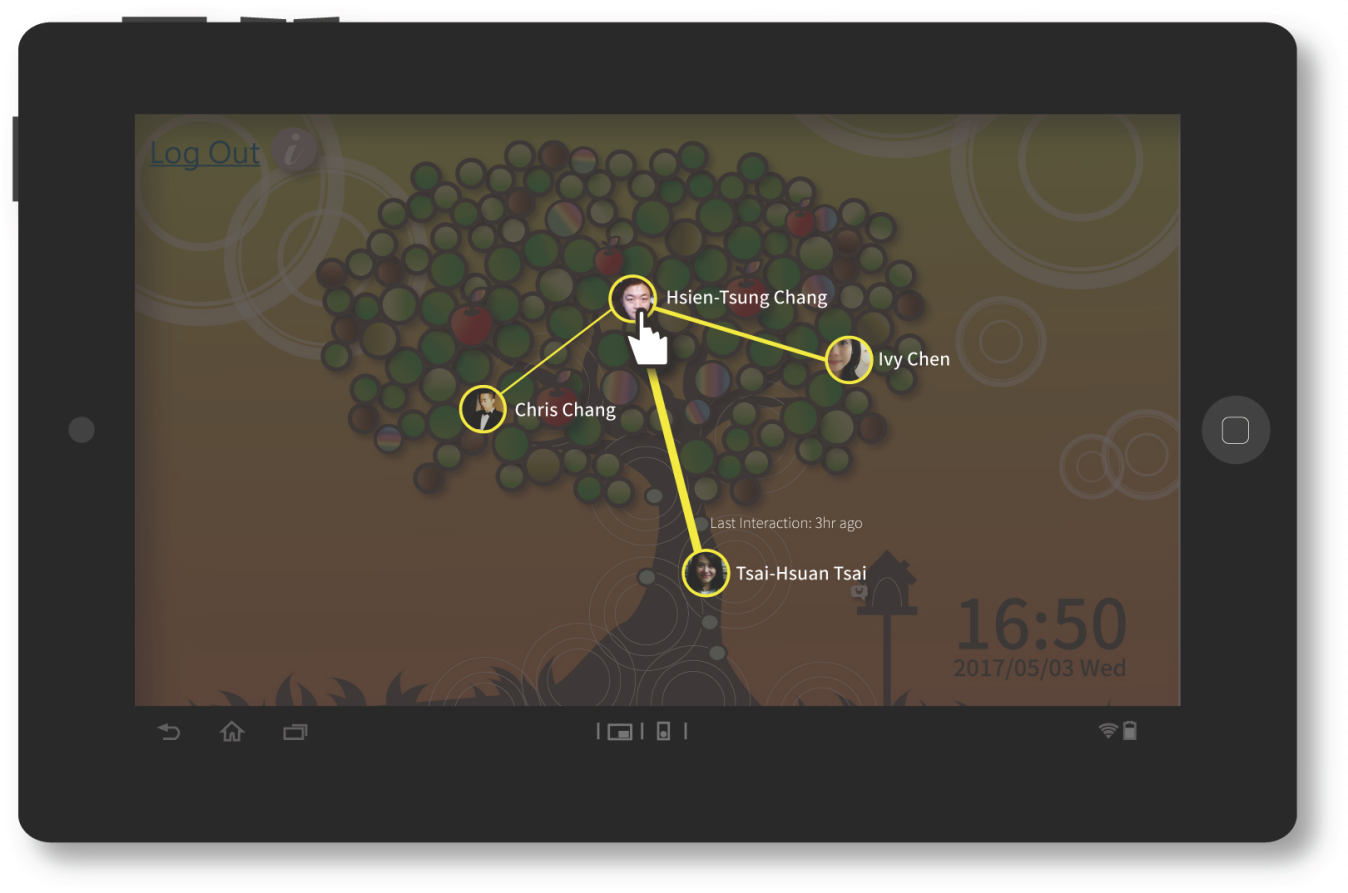
TreeIt system interface: Recent group function.

**Recent group function:** The main purpose of this function was to help promote the generation of more common links and strengthen the user’s social structure. The TreeIt system recorded the length of the interval between interactions of the user with a friend or of friends with each other. Rankings were then produced based on the number and length of the intervals. Based on photos, check-ins and other related status records, friends who had shorter interaction intervals, a higher frequency of interaction in the last two weeks and reciprocal connecting interactions with strong social intensity (reciprocally appearing on each other’s TreeIt tree) were grouped into one recent group. This function automatically updated to provide the list of friends in the friend group who had recently interacted most frequently with the user. In terms of user interface design, the recent group function required the user to hold the button representing a friend for a length of time, upon which the system would display the other friends who had recently interacted frequently with this particular friend at the top of the user interface. These were assigned to the same group, and connections among friends were shown by connecting lines, allowing the user to know which members of each group were currently interacting frequently (Fig 7).

**Fig 8:**
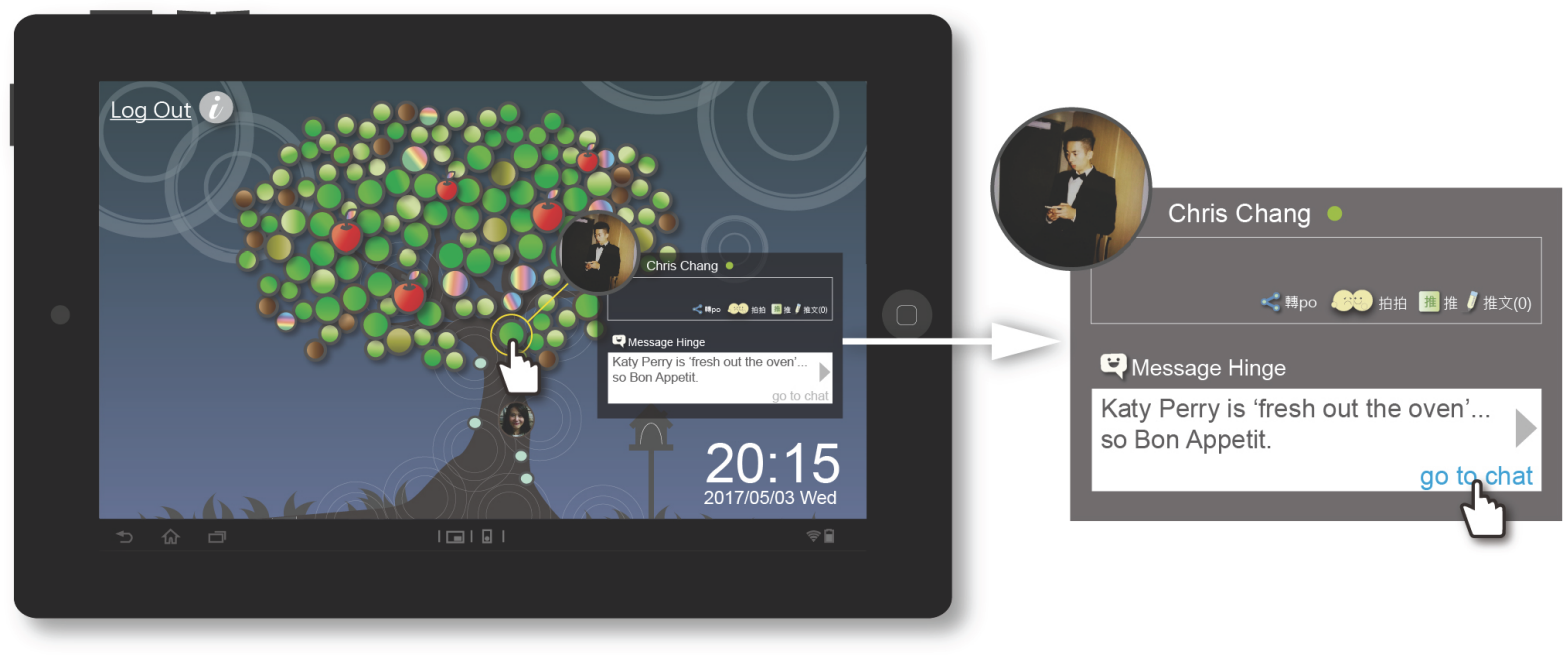
TreeIt system interface: Chat function.

**Chat function:** This function was used to shorten the distance between the user and friends through chatting and help the user enhance predictive intensity with friends. The chat function prompted the user by initiating chat topics and encouraged the user to have emotional and thoughtful interactions with friends. In terms of user interface design, the chat function required the user to take the initiative to click on a friend. The system then provided discussion topics based on common interests and trending current events, which it displayed at the bottom of the user interface. The user could then select whether to have a chat interaction on the topic provided by the system (Fig 8).

### Measurement development and statistical analysis

The survey instrument was based on the surveys developed by Zhang et al. [29] and Davis et al. [36], with modifications to match the research topic and eliminate irrelevant items. The survey asked participants to respond to a series of statements using a 5-point Likert-type scale (1 = strongly disagree to 5 = strongly agree). In accordance with the research goals and considering the appropriateness of measurement scales, this study adopted technology acceptance tests as the basis for overall pattern analysis. During the testing period, questionnaire responses were continuously added. The questionnaires were assigned a serial number, entered into a data file, and reviewed twice to correct input errors. The research hypotheses were evaluated using SEM, a powerful multivariate technique that facilitates the specification of the relationships between and among variables. For each construct shown in Fig 1, there was a single measurement model. The model describes the hypothesized problems among theoretical constructs. The structure of the diagram with a set of variables, which consists of the relationships between the latent variables and their measures, is presented as part of the model. In SEM, the measurement model is estimated using confirmatory factor analysis (CFA). In the study, LISREL8.7 was used to test the structural equation model.

## Result

### Measurement model

According to the values recommended by Nunnally et al. [66], when Cronbach's alpha is higher than 0.7 and the item-total correlation value is higher than 0.3, the questionnaire is considered to have good reliability. The composite reliability of the questionnaire is analyzed to detect internal consistency. A good composite reliability value must be higher than 0.7. As shown in Table 3, Cronbach's alpha for the variables ranged between 0.923 to 0.855, all of which are higher than the recommended value of 0.7. The item-total correlation values for the variables ranged between 0.916 and 0.749, all higher than the recommended value of 0.3. The result of the composite reliability analysis showed that the values of all the variables were higher than 0.7, indicating that overall, the questionnaire used in this study had good reliability and stable measurements. In terms of validity, construct validity is used to examine whether the variable in the questionnaire indeed measures the concept. It is divided into convergent validity and discriminant validity. Convergent validity examines the degree of convergence or connection of the items in the questionnaire with each other in terms of one variable. For convergent validity to be statistically significant, the values of composite reliability and average variance extracted (AVE) must be higher than the recommended values of 0.7 and 0.5, respectively [67]. Based on the previous analysis, the questionnaire was shown to have good composite reliability. The AVE analysis results are shown in Table 3, indicating that the values of the variables ranged between 0.7534 and 0.5536, all of which are higher than the recommended value of 0.5. Discriminant validity examines whether the scope of the constructs to be measured are different and the constructs are distinguishable. To confirm the presence of discriminant validity among the variables, the AVE of all variables must be higher than the square of all correlation coefficients. Table 4 shows that behavioral intention, perceived ease of use and perceived usefulness had good discriminant validity, whereas system support and user interface design did not. Overall, except for system support and user interface design, the variables had good discriminant validity. The mean and standard deviation of each variable are shown in Table 4. The means of all variables were higher than 4, and the standard deviations ranged between 0.560 and 0.771. Overall, the subjects showed good acceptance of TreeIt and believed it had a good user interface design.

**Table 3:** Reliability and validity of the measurement model.

Variable	Item	Cronbach’s Alpha	Factor Loading	Composite Reliability	Average Variance Extracted	Item-Total Correlation
**Behavioral Intention**	BI12	0.923	0.89	0.9241	0.7534	0.916
	BI13		0.88			0.912
	BI14		0.85			0.896
	BI15		0.85			0.886
**Perceived Usefulness**	PU1	0.912	0.87	0.9124	0.6759	0.876
	PU2		0.86			0.870
	PU3		0.80			0.860
	PU4		0.77			0.838
	PU5		0.84			0.874
**Perceived** **Ease of use**	PEOU1	0.905	0.85	0.9065	0.6201	0.860
	PEOU2		0.78			0.842
	PEOU3		0.76			0.825
	PEOU4		0.74			0.780
	PEOU5		0.70			0.756
	PEOU6		0.86			0.873
**System Support**	H7	0.855	0.61	0.8611	0.5536	0.749
	H8		0.84			0.863
	H9		0.72			0.768
	H11		0.84			0.856
	H12		0.79			0.806
**User-Interface Design**	H1	0.892	0.67	0.8988	0.6426	0.763
	H2		0.82			0.862
	H6		0.92			0.913
	H10		0.90			0.903
	H13		0.70			0.755
**Navigation**	H3	0.865	0.82	0.8578	0.6016	0.915
	H4		0.71			0.860
	H5		0.93			0.870
	H14		0.69			0.768

**Table 4:** Means, standard deviations, and discriminant validity of the latent constructs.

	Mean	S.D.	Behavioral intention	Perceived usefulness	Perceived ease of use	System support	User-interface design	Navigation
**Behavioral intention**	4.020	0.771	0.7534					
**Perceived usefulness**	4.085	0.651	0.6889	0.6759				
**Perceived ease of use**	4.170	0.680	0.5476	0.5929	0.6201			
**System** **support**	4.031	0.703	0.3844	0.2401	0.2809	0.5536		
**User-interface design**	4.431	0.560	0.2809	0.3481	0.2916	0.6241	0.6426	
**Navigation**	4.164	0.690	0.4900	0.4096	0.4489	0.7225	0.7744	0.6016

The fit indices of the measurement model are shown in Table 5. Hair et al. [68] argued that seven fit indices could be used to detect measurement model fit, i.e., Chi-square/degree of freedom (X^2^/df), goodness-of-fit index (GFI), adjusted goodness-of-fit index (AGFI), normalized fit index (NFI), non-normalized fit index (NNFI), comparative fit index (CFI) and root mean square error of approximation (RESEA). The analyses of the fitness indicators of the measurement model showed that X^2^/df was 2.10, lower than the recommended value of 3.0. The values for GFI and AGFI were 0.62 and 0.57, respectively, both below the recommended value of 0.8. The NFI, NNFI and CFI were 0.92, 0.95 and 0.96, respectively, all of which are higher than the recommended value of 0.9. Lastly, the RMSEA value was 0.099, very close to the standard value of 0.08, although not in line with the value recommended in the literature. Among the seven indices of fit, four met their recommended values, and three did not. The failure of GFI and AGFI to meet the recommended indicator fit requirements was likely due to the small sample size. Sharma et al. [69] showed that GFI and AGFI were affected by sample size. Moreover, the RMSEA value was very close to the recommended value. Therefore, overall, the measurement model exhibited appropriate fitness.

**Table 5:** Fit indices for the measurement model.

Measures	Recommended Criteria	Suggested by Authors	Measurement Model
**χ2/df**	< 3.0	[70]	2.10
**GFI**	> 0.8	[71]	0.62
**AGFI**	> 0.8	[72]	0.57
**NFI**	> 0.9	[73]	0.92
**NNFI**	> 0.9	[73]	0.95
**CFI**	> 0.9	[74]	0.96
**RMSEA**	< 0.08	[74]	0.099

### Structural model

**Fig 9:**
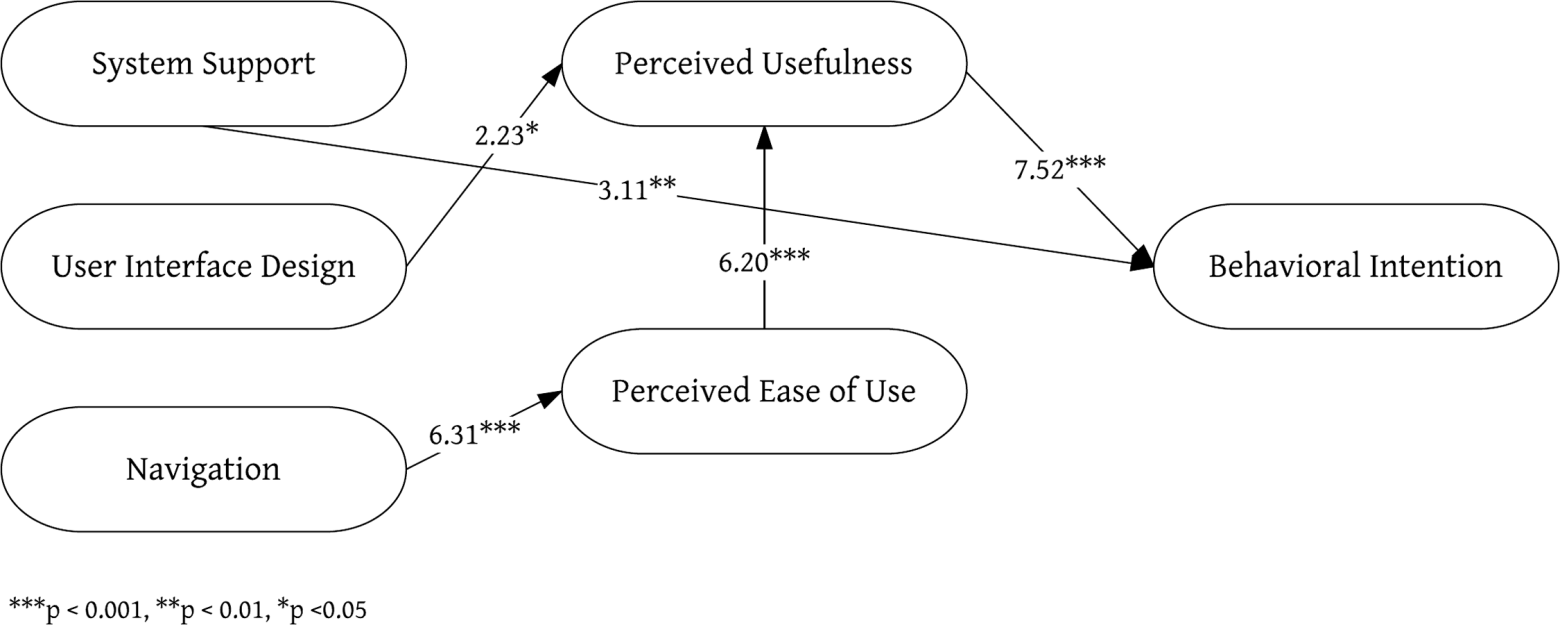
Path analysis model for the research hypotheses.

In this study, the 14 evaluation heuristics proposed by Zhang et al. were divided into three categories (system support, user interface design and navigation), and their relationships with perceived usefulness, perceived ease of use and behavioral intention of the TAM were investigated. Based on the results of path analysis, all five hypotheses were shown to be valid. The path coefficients and statistical measurements are shown in Table 6. H1 hypothesized that after using TreeIt, older adults would feel that system support and behavioral intention were positively correlated. Path analysis results showed that γ1 = 0.27 (t = 3.11, p<0.01); therefore, H1 was supported. H2 hypothesized that after using TreeIt, older adults would feel that user interface design and perceived usefulness were positively correlated. Path analysis results showed that γ2 = 0.21 (t = 2.23, p<0.05); therefore, H2 was supported. H3 hypothesized that after using TreeIt, older adults would feel that navigation and perceived ease of use were positively correlated. Path analysis results showed that γ3 = 0.65 (t = 6.31, p<0.001); therefore, H3 was supported. H4 hypothesized that after using TreeIt, older adults would feel that perceived usefulness and perceived ease of use were positively correlated. Path analysis results showed that γ4 = 0.66 (t = 6.20, p<0.001); therefore, H4 was supported. H5 hypothesized that after using TreeIt, older adults would feel that perceived usefulness and behavioral intention were positively correlated. Path analysis results showed that γ5 = 0.70 (t = 7.52, p<0.001); therefore, H5 was supported. The test results of the hypotheses are shown in Fig 9.

**Table 6:** Results of the hypothesis tests.

Exogenous Variable		Endogenous Variable	Standardized Regression Coefficient	T-value	P-value	Support
**System support**	→	Behavioral intention	0.27	3.11	**	Yes
**User-interface design**	→	Perceived usefulness	0.21	2.23	*	Yes
**Navigation**	→	Perceived ease of use	0.65	6.31	***	Yes
**Perceived ease of use**	→	Perceived usefulness	0.66	6.20	***	Yes
**Perceived usefulness**	→	Behavioral intention	0.70	7.52	***	Yes

The fit indices of the structural model are listed in Table 7. The same seven fit indices were used as for the measurement model. The fit indices analysis results for the structural model show that the X^2^/df was 2.10, lower than the recommended value of 3.0. The GF and AGFI were 0.68 and 0.63, respectively, both below the recommended value of 0.8. The NFI, NNFI and CFI were 0.92, 0.95 and 0.96, respectively, all above the recommended value of 0.9. Lastly, the RMSEA value was 0.090, very close to the standard value of 0.08, although not in line with the value recommended in the literature. Among the seven indices of fit, four met the recommended values and three did not, which is consistent with the results for the measurement model. It is postulated that the failure of GFI and AGFI to meet the recommended indicator fit requirements was likely due to the small sample size [69]. Therefore, the structural model exhibited appropriate fitness overall.

**Table 7:** Fit indices for the structural model.

Measures	Recommended Criteria	Suggested by Authors	Structural Model
**χ2/df**	< 3.0	[70]	2.10
**GFI**	> 0.8	[71]	0.68
**AGFI**	> 0.8	[72]	0.63
**NFI**	> 0.9	[73]	0.92
**NNFI**	> 0.9	[73]	0.95
**CFI**	> 0.9	[74]	0.96
**RMSEA**	< 0.08	[74]	0.090

## Discussion

In this study, we investigated TreeIt, a social application specifically developed for older adults that contained seven social promotion mechanisms. By using the system, TreeIt allows older adults to increase social connection, maintain intensity of social connection and strengthen social experience. To investigate the effect of the user interface usability on older adults’ intention and attitude related to using the system, 14 usability heuristics proposed by Zhang et al. [29] were used to evaluate user interface usability. The heuristics were grouped into three categories (system support, user interface design and navigation). The behavior of older adults with respect to using the TreeIt system was assessed by combining the heuristics with the TAM. The results showed that the five hypotheses proposed in this study were all valid. In terms of system support, the results of this study validated the H1 hypothesis that system support significantly affects behavioral intention. After the elderly subjects operated the TreeIt system, many thought that TreeIt’s social display function and recent group function helped remind them of their friends’ social status and expressed a willingness to maintain social relations with friends via the TreeIt system. Moreover, a number of subjects claimed that the hot-topic function helped them pay attention to friends’ messages. The users noted that the user interface design made it easy to operate the TreeIt system without being prone to making operational errors. Even in case of errors, the users were easily able to fix them by themselves. In terms of user interface design, the relationship between user interface design and perceived usefulness was validated. The TreeIt system used a tree to represent users and leaves to represent friends’ social status. The number of leaves represented the number of friends, whereas the size of a leaf represented the intensity of the social relationship with a particular friend. Many subjects noted that the analogy was appropriate and clear. The visual appearance of the system, namely, layout, color schemes, icons, buttons, etc., also conformed to the concept of the tree. Regarding the mood display/ emotional feedback function, when one friend accumulated or maintained a certain level of emotional feedback, the color of the leaf changed or remained the same to indicate the friend’s positive or negative mood. The subjects claimed that clear system user interface design helped them maintain or strengthen their social relations with friends. In the navigation section, the results of the study were consistent with those of Ramayah [61], indicating that navigation significantly affected perceived ease of use. As the user interface design of the TreeIt system was fairly simple and only contained three operation layers at most, navigation was easy for the users. The majority of the subjects indicated that the operation of the TreeIt system did not impose too large of a cognitive load.

Lastly, in terms of confirming the intrinsic relationship among technology acceptance factors, the results of this study showed that perceived ease of use directly affected perceived usefulness. Behavioral intention was also directly affected by perceived usefulness, consistent with the findings of Rauniar et al. [40], who found that in using social applications, if users perceived a system as easier to operate, they would find it more useful and be more likely to use the social application.

## Conclusion

Regarding the validation of system usability, previous studies have investigated the relationship between quality and usability or the relationship between user interface and perceived ease of use or perceived usefulness. However, in those studies, the definitions of usability and user interface were too broad and only portions of Nielsen’s usability heuristics were applied. Because there have been no detailed investigations on the effect of system user interface usability on older adults’ attitude and intention to use SNSs, this study divided the 14 heuristics proposed by Zhang et al. [29] into three categories (system support, user interface design and navigation) and integrated them into the TAM. The results showed that system support and perceived usefulness had a significant effect on behavioral intention. Furthermore, user interface design and perceived ease of use were positively correlated with perceived usefulness. Navigation also had a positive correlation with perceived ease of use. These findings can provide valuable suggestions for designing social applications’ user interface for older adults in the future. However, this study also has some limitations. First, to maintain social intensity, the system automatically filtered out friends with whom the user had weak connections and limited users to 150 friends. However, users might found it difficult to maintain their social connections with friends who were initially filtered out. As a result, we suggest that the system allow users to manually search for friends who were previously filtered out. Second, the study was conducted by inviting subjects to operate seven functions in an experimental fashion. The subjects were unable to interact with their real friends through the system. Therefore, it is recommended that a long-term study be conducted to examine elderly subjects’ real interactions with their friends through the TreeIt system. Finally, the main purpose of this study was to understand whether user interface usability affects users’ acceptance of new technology. In future studies, the effect of other internal or external factors such as gender, subjective norms, and self-efficacy on older adults’ use intention and acceptance of social applications should also be investigated.

## Supporting information

S1 File Appendix 1. Treeit heuristic evaluation.
(PDF)

S2 File Appendix 2. TreeIt technology acceptance model questionnaire.
(PDF)

S3 File Data for PLOS ONE TreeIt. (XLSX)

## References

[1] Seeman TE (1996) Social ties and health: The benefits of social integration. Annals of epidemiology 6: 442–451. 8915476 10.1016/s1047-2797(96)00095-6

[2] Bondevik M, Skogstad A (1998) The oldest old, ADL, social network, and loneliness. Western Journal of Nursing Research 20: 325–343. doi: 10.1177/019394599802000305 9615601 10.1177/019394599802000305

[3] Francis D (1991) Friends from the workplace. Growing old in America: 465–480.

[4] De Vogli R, Chandola T, Marmot MG (2007) Negative aspects of close relationships and heart disease. Archives of Internal Medicine 167: 1951–1957. doi: 10.1001/archinte.167.18.1951 17923594 10.1001/archinte.167.18.1951

[5] Cattan M, White M, Bond J, Learmouth A (2005) Preventing social isolation and loneliness among older people: a systematic review of health promotion interventions. Ageing and society 25: 41–67. 10.7748/nop.17.1.40.s11 27736564

[6] Czaja SJ, Guerrier JH, Nair SN, Landauer TK (1993) Computer communication as an aid to independence for older adults. Behaviour & Information Technology 12: 197–207.

[7] Bennett DA, Schneider JA, Tang Y, Arnold SE, Wilson RS (2006) The effect of social networks on the relation between Alzheimer's disease pathology and level of cognitive function in old people: a longitudinal cohort study. The Lancet Neurology 5: 406–412. doi: 10.1016/S1474-4422(06)70417-3 16632311 10.1016/S1474-4422(06)70417-3

[8] Glass TA, de Leon CM, Marottoli RA, Berkman LF (1999) Population based study of social and productive activities as predictors of survival among elderly Americans. Bmj 319: 478–483. 10454399 10.1136/bmj.319.7208.478 PMC28199

[9] Stevens N (2001) Combating loneliness: a friendship enrichment programme for older women. Ageing and Society 21: 183–202.

[10] Bedford VH (1998) Sibling relationship troubles and well-being in middle and old age. Family Relations: 369–376.

[11] Hartup WW, Stevens N (1997) Friendships and adaptation in the life course. Psychological bulletin 121: 355.

[12] Ellison N, Steinfield C, Lampe C (2006) Spatially bounded online social networks and social capital. International Communication Association 36.

[13] Duggan M, Ellison NB, Lampe C, Lenhart A, Madden M (2015) Social Media Update 2014. PewResearchCenter.

[14] Brenner J, Smith A (2013) 72% of Online Adults are Social Networking Site Users. PewResearchCenter.

[15] Zickuhr K, Madden M (2012) Older adults and internet use. PewResearchCenter.

[16] PawPawMail.com (2011).

[17] Keep In Touch (2012).

[18] Granovetter MS (1973) The strength of weak ties. American journal of sociology: 1360–1380.

[19] Gilbert E, Karahalios K (2009) Predicting tie strength with social media. Proceedings of the SIGCHI Conference on Human Factors in Computing Systems. Boston, MA, USA: ACM. pp. 211–220.

[20] Dunbar RIM (1992) Neocortex size as a constraint on group size in primates. Journal of Human Evolution 22: 469–493.

[21] Stone D, Jarrett C, Woodroffe M, Minocha S (2005) User interface design and evaluation: Morgan Kaufmann.

[22] Jaspers MW (2009) A comparison of usability methods for testing interactive health technologies: methodological aspects and empirical evidence. International journal of medical informatics 78: 340–353. doi: 10.1016/j.ijmedinf.2008.10.002 19046928 10.1016/j.ijmedinf.2008.10.002

[23] Tan W-s, Liu D, Bishu R (2009) Web evaluation: Heuristic evaluation vs. user testing. International Journal of Industrial Ergonomics 39: 621–627.

[24] Nielsen J (1995) Ten Usability Heuristics. Nielsen Norman Group.

[25] Atashi A, Khajouei R, Azizi A, Dadashi A (2016) User Interface Problems of a Nationwide Inpatient Information System: A Heuristic Evaluation. Applied clinical informatics 7: 89–100. doi: 10.4338/ACI-2015-07-RA-0086 27081409 10.4338/ACI-2015-07-RA-0086 PMC4817337

[26] Choi J, Bakken S (2010) Web-based education for low-literate parents in Neonatal Intensive Care Unit: Development of a website and heuristic evaluation and usability testing. International Journal of Medical Informatics 79: 565–575. doi: 10.1016/j.ijmedinf.2010.05.001 20617546 10.1016/j.ijmedinf.2010.05.001 PMC2956000

[27] Hvannberg ET, Law EL-C, Lérusdóttir MK (2007) Heuristic evaluation: Comparing ways of finding and reporting usability problems. Interacting with computers 19: 225–240.

[28] Schneiderman B, Plaisant C (1998) Designing the user interface Don Mills, Ontario: Addison Wesley.

[29] Zhang J, Johnson TR, Patel VL, Paige DL, Kubose T (2003) Using usability heuristics to evaluate patient safety of medical devices. Journal of biomedical informatics 36: 23–30. 14552844 10.1016/s1532-0464(03)00060-1

[30] Allen M, Currie LM, Bakken S, Patel VL, Cimino JJ (2006) Heuristic evaluation of paper-based Web pages: a simplified inspection usability methodology. Journal of biomedical informatics 39: 412–423. doi: 10.1016/j.jbi.2005.10.004 16321575 10.1016/j.jbi.2005.10.004

[31] Chan AJ, Islam MK, Rosewall T, Jaffray DA, Easty AC, et al (2012) Applying usability heuristics to radiotherapy systems. Radiotherapy and Oncology 102: 142–147. doi: 10.1016/j.radonc.2011.05.077 21733590 10.1016/j.radonc.2011.05.077

[32] Tang Z, Johnson TR, Tindall RD, Zhang J (2006) Applying heuristic evaluation to improve the usability of a telemedicine system. Telemedicine Journal & E-Health 12: 24–34. 16478410 10.1089/tmj.2006.12.24

[33] Al-Badi AH, Okam MO, Al Roobaea R, Mayhew PJ (2013) Improving usability of social networking systems: a case study of LinkedIn. Journal of Internet Social Networking & Virtual Communities 2013: 1.

[34] Ashraf A, Raza A (2013) Heuristic Evaluation of Social Websites: For Blind People. International Journal of Computer and Communication Engineering 2: 711.

[35] Castilla D, Garcia-Palacios A, Breton-Lopez J, Miralles I, BañOs RM, et al (2013) Process of design and usability evaluation of a telepsychology web and virtual reality system for the elderly: Butler. International Journal of Human-Computer Studies 71: 350–362.

[36] Davis FD, Bagozzi RP, Warshaw PR (1989) User acceptance of computer technology: a comparison of two theoretical models. Management science 35: 982–1003.

[37] Yang S-C, Lin C-H (2011) Factors affecting the intention to use Facebook to support problem-based learning among employees in a Taiwanese manufacturing company. African Journal of Business Management 5: 9014.

[38] Hu T, Poston RS, Kettinger WJ (2011) Nonadopters of online social network services: Is it easy to have fun yet. Communications of the Association for Information Systems 29: 441–458.

[39] Choi G, Chung H (2013) Applying the technology acceptance model to social networking sites (SNS): Impact of subjective norm and social capital on the acceptance of SNS. International Journal of Human-Computer Interaction 29: 619–628.

[40] Rauniar R, Rawski G, Yang J, Johnson B (2014) Technology acceptance model (TAM) and social media usage: an empirical study on Facebook. Journal of Enterprise Information Management 27: 6–30.

[41] Braun MT (2013) Obstacles to social networking website use among older adults. Computers in Human Behavior 29: 673–680.

[42] Pan S, Jordan-Marsh M (2010) Internet use intention and adoption among Chinese older adults: From the expanded technology acceptance model perspective. Computers in human behavior 26: 1111–1119.

[43] Tsai T-H, Chang H-T, Ho Y-L (2016) Perceptions of a Specific Family Communication Application among Grandparents and Grandchildren: An Extension of the Technology Acceptance Model. PloS one 11: e0156680 doi: 10.1371/journal.pone.0156680 27270915 10.1371/journal.pone.0156680 PMC4896451

[44] Nikov A, Zaim S, Oztekin A (2006) Usability evaluation of web services by structural equation modeling University of West Indies: Trinidad.

[45] Oztekin A, Nikov A, Zaim S (2009) UWIS: An assessment methodology for usability of web-based information systems. Journal of Systems and Software 82: 2038–2050.

[46] Oztekin A, Kong ZJ, Uysal O (2010) UseLearn: A novel checklist and usability evaluation method for eLearning systems by criticality metric analysis. International Journal of Industrial Ergonomics 40: 455–469.

[47] Calisir F, Calisir F (2004) The relation of interface usability characteristics, perceived usefulness, and perceived ease of use to end-user satisfaction with enterprise resource planning (ERP) systems. Computers in human behavior 20: 505–515.

[48] Cho V, Cheng TE, Lai WJ (2009) The role of perceived user-interface design in continued usage intention of self-paced e-learning tools. Computers & Education 53: 216–227.

[49] González M, Masip L, Granollers A, Oliva M (2009) Quantitative analysis in a heuristic evaluation experiment. Advances in Engineering Software 40: 1271–1278.

[50] Chang SJ, Im E-O (2014) A path analysis of Internet health information seeking behaviors among older adults. Geriatric Nursing 35: 137–141. doi: 10.1016/j.gerinurse.2013.11.005 24332965 10.1016/j.gerinurse.2013.11.005

[51] Guo X, Sun Y, Wang N, Peng Z, Yan Z (2013) The dark side of elderly acceptance of preventive mobile health services in China. Electronic Markets 23: 49–61.

[52] Renaud K, Van Biljon J. Predicting technology acceptance and adoption by the elderly: a qualitative study; 2008 ACM pp. 210–219.

[53] Igbaria M, Iivari J (1995) The effects of self-efficacy on computer usage. Omega 23: 587–605.

[54] Wilkinson A, Forbes A, Bloomfield J, Gee CF (2004) An exploration of four web-based open and flexible learning modules in post-registration nurse education. International journal of nursing studies 41: 411–424. doi: 10.1016/j.ijnurstu.2003.11.001 15050852 10.1016/j.ijnurstu.2003.11.001

[55] Branscomb LM, Thomas JC (1984) Ease of use: a system design challenge. IBM Systems Journal 23: 224–235.

[56] Jeong H (2011) An investigation of user perceptions and behavioral intentions towards the e-library. Library Collections, Acquisitions, and Technical Services 35: 45–60.

[57] Graham M, Hannigan K, Curran P (2005) Imagine: Visual design in first-year composition. Journal of Visual Literacy 25: 21–40.

[58] Hong W, Thong JY, Wai-Man Wong K-YT (2002) Determinants of user acceptance of digital libraries: an empirical examination of individual differences and system characteristics. Journal of Management Information Systems 18: 97–124.

[59] Dillon A (2000) Spatial‐semantics: How users derive shape from information space. Journal of the American Society for Information Science 51: 521–528.

[60] Marchionini G, Plaisant C, Komlodi A (1998) Interfaces and tools for the Library of Congress national digital library program. Information processing & management 34: 535–555.

[61] Ramayah T (2006) Interface characteristics, perceived ease of use and intention to use an online library in Malaysia. Information Development 22: 123–133.

[62] Venkatesh V, Davis FD (2000) A theoretical extension of the technology acceptance model: Four longitudinal field studies. Management science 46: 186–204.

[63] King WR, He J (2006) A meta-analysis of the technology acceptance model. Information & management 43: 740–755.

[64] Folstein MF, Folstein SE, McHugh PR (1975) “Mini-mental state”: a practical method for grading the cognitive state of patients for the clinician. Journal of psychiatric research 12: 189–198. 1202204 10.1016/0022-3956(75)90026-6

[65] Gilbert E. Predicting tie strength in a new medium; 2012 ACM pp. 1047–1056.

[66] Nunnally JC, Bernstein IH, Berge JMt (1967) Psychometric theory: JSTOR.

[67] McCloskey DW (2006) The importance of ease of use, usefulness, and trust to online consumers: An examination of the technology acceptance model with older consumers. Journal of Organizational and End User Computing 18: 47.

[68] Hair JF, Anderson RE, Tatham RL, William C (1998) Black (1998), Multivariate data analysis. Upper Saddle River, NJ: Prentice Hall.

[69] Sharma S, Mukherjee S, Kumar A, Dillon WR (2005) A simulation study to investigate the use of cutoff values for assessing model fit in covariance structure models. Journal of Business Research 58: 935–943.

[70] Bentler PM, Bonett DG (1980) Significance tests and goodness of fit in the analysis of covariance structures. Psychological bulletin 88: 588.

[71] Seyal AH, Rahman MNA, Rahim MM (2002) Determinants of academic use of the Internet: a structural equation model. Behaviour & Information Technology 21: 71–86.

[72] Scott JE (1995) The measurement of information systems effectiveness: evaluating a measuring instrument. ACM SIGMIS Database 26: 43–61.

[73] Hair JFJF (1979) Multivariate data analysis: with readings.

[74] Bagozzi RP, Yi Y (1988) On the evaluation of structural equation models. Journal of the academy of marketing science 16: 74–94.

