# Supplementary material for: Determinants of user acceptance of a specific social platform for older adults: An empirical examination of user interface characteristics and behavioral intention
Source: PLoS One. 2017 Aug 24;12(8):e0180102. doi: 10.1371/journal.pone.0180102 (PMC5570369; doi:10.1371/journal.pone.0180102)
Supplement: S1 File — Treeit heuristic evaluation. (PDF) [file pone.0180102.s001.pdf]

Appendix 1: Treeit Heuristic Evaluation

H1. Consistency

Consistency and standards. Users should not have to wonder whether different words, situations, or actions mean the same thing. Standards and conventions in product design should be followed.

Item	Comments		Very unsatisfied	Unsatisfied	Neutral	Satisfied	Very satisfied
	Advantage	Disadvantage	1	2	3	4	5
Sequences of actions (skill acquisition).			☐	☐	☐	☐	☐
The system uses different colors to categorize different items.			☐	☐	☐	☐	☐
Layout and position (spatial consistency).			☐	☐	☐	☐	☐
Font, capitalization (levels of organization).			☐	☐	☐	☐	☐
Terminology (delete, del, remove, rm) and language (words, phrases).			☐	☐	☐	☐	☐
Standards (e.g., blue underlined text for unvisited hyperlinks).			☐	☐	☐	☐	☐

H2. Visibility:

Visibility of system state. Users should be informed about what is going on with the system through appropriate feedback and display of information.

Item	Comments	Very unsatisfied	Unsatisfied	Neutral	Satisfied	Very satisfied

	Advantage	Disadvantage	1	2	3	4	5
The interface shows the current state of the system.			☐	☐	☐	☐	☐
The interface shows what can be done in the current state.			☐	☐	☐	☐	☐
The interface shows where the user can go (e.g., providing a hyperlink).			☐	☐	☐	☐	☐
The interface can show what change is made after an action.			☐	☐	☐	☐	☐

H3. Match
Match between system and world. The image of the system perceived by users should match the model the users have about the system.

Item	Comments		Very unsatisfied	Unsatisfied	Neutral	Satisfied	Very satisfied
	Advantage	Disadvantage	1	2	3	4	5
User model matches system image.			☐	☐	☐	☐	☐
Actions provided by the system match the actions performed by users.			☐	☐	☐	☐	☐
Objects in the system match the objects of the task.			☐	☐	☐	☐	☐

H4. Minimalist
Any extraneous information is a distraction and a slow-down.

Item	Comments	Very unsatisfied	Unsatisfied	Neutral	Satisfied	Very satisfied

	Advantage	Disadvantage	1	2	3	4	5
The interface provides concise information. Less is more.			☐	☐	☐	☐	☐
Though the system provides simple information, it is not equivalent to abstract and general.			☐	☐	☐	☐	☐
The system provides simple information and makes it efficient to operate.			☐	☐	☐	☐	☐
Progressive levels of details.			☐	☐	☐	☐	☐

H5. Memory

Minimize memory load. Users should not be required to memorize a lot of information to carry out tasks. Memory load reduces users' capacity to carry out the main tasks.

Item	Comments		Very unsatisfied	Unsatisfied	Neutral	Satisfied	Very satisfied
	Advantage	Disadvantage	1	2	3	4	5
Recognition vs. recall (e.g., menu vs. commands).			☐	☐	☐	☐	☐
Externalize information through visualization.			☐	☐	☐	☐	☐
The ways to execute the system follow perceptual procedures.			☐	☐	☐	☐	☐
The ways to execute the system follow a hierarchical structure.			☐	☐	☐	☐	☐

When executing the system, the system initially follows the default values of the setting.			☐	☐	☐	☐	☐
Concrete examples (DD/MM/YY, e.g., 10/20/99).			☐	☐	☐	☐	☐
Generic rules and actions (e.g., drag objects).			☐	☐	☐	☐	☐

H6. Feedback

Informative feedback. Users should be given prompt and informative feedback about their actions.

Item	Comments		Very unsatisfied	Unsatisfied	Neutral	Satisfied	Very satisfied
	Advantage	Disadvantage	1	2	3	4	5
Information that can be directly perceived, interpreted, and evaluated.			☐	☐	☐	☐	☐
The levels of feedback are the same for novice and expert.			☐	☐	☐	☐	☐
Concrete and specific, not abstract and general.			☐	☐	☐	☐	☐
The response time is reasonable and not too long.			☐	☐	☐	☐	☐

H7. Flexibility

Flexibility and efficiency. Users always learn, and users are always different. Give users the flexibility of creating customization and shortcuts to accelerate their performance.

Item	Comments		Very unsatisfied	Unsatisfied	Neutral	Satisfied	Very satisfied

	Advantage	Disadvantage	1	2	3	4	5
Shortcuts for experienced users.			☐	☐	☐	☐	☐
Shortcuts or macros for frequently used operations.			☐	☐	☐	☐	☐
Skill acquisition through chunking.			☐	☐	☐	☐	☐

H8. Message
Good error messages. The messages should be informative enough so that users can understand the nature of errors, learn from errors, and recover from errors.

Item	Comments		Very unsatisfied	Unsatisfied	Neutral	Satisfied	Very satisfied
	Advantage	Disadvantage	1	2	3	4	5
Phrased in clear language, avoiding obscure codes. Example of obscure code: “system crashed, error code 147.’’			☐	☐	☐	☐	☐
Precise, not vague or general. Example of general comment: “Cannot open document.”			☐	☐	☐	☐	☐
The system provides constructive messages when error happens.			☐	☐	☐	☐	☐
The system provides polite messages when errors occur. Examples of impolite message: “illegal user action,” “job aborted,” “system was crashed,” “fatal error,” etc.			☐	☐	☐	☐	☐

H9. Error
Prevent errors. It is always better to design interfaces that prevent errors from happening in the first place.

Item	Comments		Very unsatisfied	Unsatisfied	Neutral	Satisfied	Very satisfied
	Advantage	Disadvantage	1	2	3	4	5
Interfaces that make errors impossible.			☐	☐	☐	☐	☐
The system provides avoid modes or uses informative feedback, e.g., different sounds.			☐	☐	☐	☐	☐
Execution error vs. evaluation error.			☐	☐	☐	☐	☐
The system can prevent various types of slips and mistakes.			☐	☐	☐	☐	☐

H10. Closure

Clear closure. Every task has a beginning and an end. Users should be clearly notified about the completion of a task.

Item	Comments		Very unsatisfied	Unsatisfied	Neutral	Satisfied	Very satisfied
	Advantage	Disadvantage	1	2	3	4	5
Clear beginning, middle, and end.			☐	☐	☐	☐	☐
Complete 7-stages of actions.			☐	☐	☐	☐	☐
Clear feedback to indicate goals are achieved and current stacks of goals can be released. Examples of good closures include many dialogues.			☐	☐	☐	☐	☐

H11. Undo
Reversible actions. Users should be allowed to recover from errors. Reversible actions also encourage exploratory learning.

Item	Comments		Very unsatisfied	Unsatisfied	Neutral	Satisfied	Very satisfied
	Advantage	Disadvantage	1	2	3	4	5
Each single step of a function can be repeated and allow for returning to previous steps.			☐	☐	☐	☐	☐
Each function has multiple steps and users can return to previous steps.			☐	☐	☐	☐	☐
The system encourages exploratory learning.			☐	☐	☐	☐	☐
The system can prevent serious errors.			☐	☐	☐	☐	☐

H12. Language
Use users' language. The language should always be presented in a form that is understandable to the intended users.

Item	Comments		Very unsatisfied	Unsatisfied	Neutral	Satisfied	Very satisfied
	Advantage	Disadvantage	1	2	3	4	5
Use standard meanings of words.			☐	☐	☐	☐	☐
Specialized language for specialized groups.			☐	☐	☐	☐	☐
User can define aliases.			☐	☐	☐	☐	☐

**H13. Control**<br>Users in control. Do not give users the impression that they are controlled by the systems.							
Item	Comments		Very unsatisfied	Unsatisfied	Neutral	Satisfied	Very satisfied
	Advantage	Disadvantage	1	2	3	4	5
Users are initiators of actors, not responders to actions.			☐	☐	☐	☐	☐
Avoid surprising actions, unexpected outcomes, tedious sequences of actions, etc.			☐	☐	☐	☐	☐

**H14. Document**<br>Help and documentation. Always provide help when needed.							
Item	Comments		Very unsatisfied	Unsatisfied	Neutral	Satisfied	Very satisfied
	Advantage	Disadvantage	1	2	3	4	5
The system provides context-sensitive help.			☐	☐	☐	☐	☐
Four types of help: task-oriented, alphabetically ordered, semantically organized, search.			☐	☐	☐	☐	☐
The system has help embedded in contents.			☐	☐	☐	☐	☐
