# Supplementary material for: Determinants of user acceptance of a specific social platform for older adults: An empirical examination of user interface characteristics and behavioral intention
Source: PLoS One. 2017 Aug 24;12(8):e0180102. doi: 10.1371/journal.pone.0180102 (PMC5570369; doi:10.1371/journal.pone.0180102)
Supplement: S2 File — TreeIt technology acceptance model questionnaire. (PDF) [file pone.0180102.s002.pdf]

Appendix 2: TreeIt Technology Acceptance Model Questionnaire

Researcher:

Date:

Section One: Background Information:

[1] Gender ☐ Male ☐ Female

[2] Age: ______________ years old

[3] Educational Level:

☐ Elementary School ☐ Junior High ☐ Senior High ☐ Bachelor's Degree

☐ Graduate Degree

[4] Do you have experience in using social network sites?

☐ Yes (Go to question 5 and 6) ☐ No (Please go directly to Section Two)

[5] Your preference in using social network sites (multiple choices):

☐ Facebook ☐ Twitter ☐ google+ ☐ LinkedIn ☐ Sina Weibo ☐ Others_________

[6] Your habits when using social network sites (multiple choices):

☐ Facebook ☐ Twitter ☐ google+ ☐ LinkedIn ☐ Sina Weibo ☐ Others_________

Section Two: TAM Questions:

	Strongly disagree	Disagree	Neutral	Agree	Strongly agree
Using the TreeIt system helps me understand my social status.	☐	☐	☐	☐	☐
Using the TreeIt system helps me quickly analyze my social status.	☐	☐	☐	☐	☐
Compared to other SNSs, the TreeIt system provides more complete inspection of my social status.	☐	☐	☐	☐	☐

Compared to other SNSs, it is more convenient to use the TreeIt system.	☐	☐	☐	☐	☐
Overall, I think the TreeIt system is useful for me.	☐	☐	☐	☐	☐
I find the TreeIt system to be easy to use.	☐	☐	☐	☐	☐
I find the TreeIt system easy to learn.	☐	☐	☐	☐	☐
My interaction with the TreeIt system is clear and understandable.	☐	☐	☐	☐	☐
Compared to other SNSs, the TreeIt system has a clearer and easier operating interface.	☐	☐	☐	☐	☐
Compared to other SNSs, the TreeIt system provides a more humanized operating interface.	☐	☐	☐	☐	☐
Overall, I think the TreeIt system is easy to use.	☐	☐	☐	☐	☐
To inspect my social status in the future, I am willing to continue using the TreeIt system.	☐	☐	☐	☐	☐
I plan to use the TreeIt system to inspect my social status.	☐	☐	☐	☐	☐
I will recommend the TreeIt system to my family and friends.	☐	☐	☐	☐	☐
Overall, I have a high intention to use the TreeIt system.	☐	☐	☐	☐	☐

Thank you for taking time to participate in our survey. We truly value the information you have provided.
